# Self-Assembled Nano-PROTACs for Targeted Protein Degradation in Cancer Therapy

**DOI:** 10.3390/molecules31214024

**Published:** 2026-11-08

**Authors:** Jiahui Dai, Yang Liu, Xueya Wang, Xinping Zhou, Changrui Xie, Yong Jin

**Affiliations:** School of Pharmacy, Anhui Medical University, Hefei 231200, China; 2645010852@stu.ahmu.edu.cn (J.D.); 2645010876@stu.ahmu.edu.cn (Y.L.); 2545011079@stu.ahmu.edu.cn (X.W.); 2645010873@stu.ahmu.edu.cn (X.Z.); 2645010877@stu.ahmu.edu.cn (C.X.)

**Keywords:** self-assembled Nano-PROTACs, targeted protein degradation, cancer therapy, stimuli-responsive, clinical translation

## Abstract

Proteolysis-targeting chimeras (PROTACs) induce target protein ubiquitination and proteasomal degradation via recruitment of E3 ubiquitin ligases, enabling event-driven intervention against “undruggable” targets, drug-resistant proteins, and non-catalytic scaffolding functions. Conventional PROTACs, however, face limitations including high molecular weight, poor solubility and membrane permeability, unfavorable pharmacokinetics, limited tissue selectivity, and the hook effect. Self-assembled Nano-PROTACs allow direct nanostructure formation from intact PROTACs, prodrugs, or functional modules, and permit spatiotemporal activation by tumor microenvironment or exogenous stimuli. This review systematically examines four assembly paradigms—carrier-free co-assembly, amphiphilic conjugates, peptide- and nucleic acid-programmed assembly, and split intracellular systems—and positions stimuli-responsiveness as a cross-architectural activation hallmark. Representative studies across cancer types are categorized to analyze how self-assembled Nano-PROTACs intervene in key oncogenic processes, with comparisons of target selectivity, activation mechanisms, degradation evidence, and antitumor efficacy. Finally, clinical translation requirements are discussed regarding structure–assembly–degradation relationships, safety, quality control, and scalable manufacturing, aiming to facilitate further research and clinical development of self-assembled Nano-PROTACs in cancer therapy.

## 1. Introduction

### 1.1. Cancer Disease Burden and Therapeutic Challenges

Malignant tumors remain one of the leading causes of death worldwide. The latest GLOBOCAN 2024 estimates report approximately 20.6 million new cancer cases and 9.8 million deaths globally in 2024, and population aging together with exposure to risk factors is expected to further increase the disease burden [1]. Cancer is not driven by aberrations in a single pathway, but rather by a constellation of hallmarks—sustained proliferation, evasion of growth suppression, resistance to cell death, metabolic reprogramming, and immune evasion. Genomic instability and the heterogeneity of the tumor microenvironment further amplify both inter-patient and intra-lesional variability [2].

Current cancer treatment comprises surgery, radiotherapy, chemotherapy, molecular targeted therapy, endocrine therapy, antibody-based drugs, and immunotherapy; however, each modality has distinct applicability boundaries. Conventional cytotoxic therapy is frequently compromised by dose-limiting toxicities and drug resistance. Although immunotherapy can induce durable responses, its efficacy is constrained by insufficient immunogenicity, an immunosuppressive tumor microenvironment, and acquired resistance. Nanoparticle-mediated delivery can improve the exposure of certain drugs, yet interpatient delivery variability and solid tumor heterogeneity still limit consistent clinical benefit [3,4]. At the molecular level, tumor initiation and progression rely on a series of abnormally expressed, mutated, or constitutively activated key proteins; interfering with these pathogenic proteins can effectively disrupt tumor-associated signaling networks. However, conventional targeted drugs primarily rely on sustained binding to druggable pockets to exert their effects through inhibition of catalytic or functional activity. Consequently, they often fail to effectively intervene in pathogenic processes driven by proteins lacking well-defined binding pockets, as well as those mediated by non-catalytic scaffolding functions or protein–protein interactions. In contrast to merely inhibiting protein function, direct reduction or clearance of pathogenic proteins can simultaneously abolish both their catalytic and non-catalytic functions, and is expected to expand the target landscape beyond the reach of conventional drugs. Based on this rationale, targeted protein degradation (TPD) technologies, represented by proteolysis-targeting chimeras (PROTACs), have gradually emerged as a critical research focus in tumor-targeted therapy.

### 1.2. Targeted Protein Degradation and PROTAC Technology

PROTACs induce targeted protein degradation in an event-driven manner, simultaneously abolishing both catalytic and non-catalytic functions under appropriate conditions, and offering a new interventional avenue for drug-resistant proteins and targets lacking druggable pockets [5,6,7,8].

**Figure 1:**
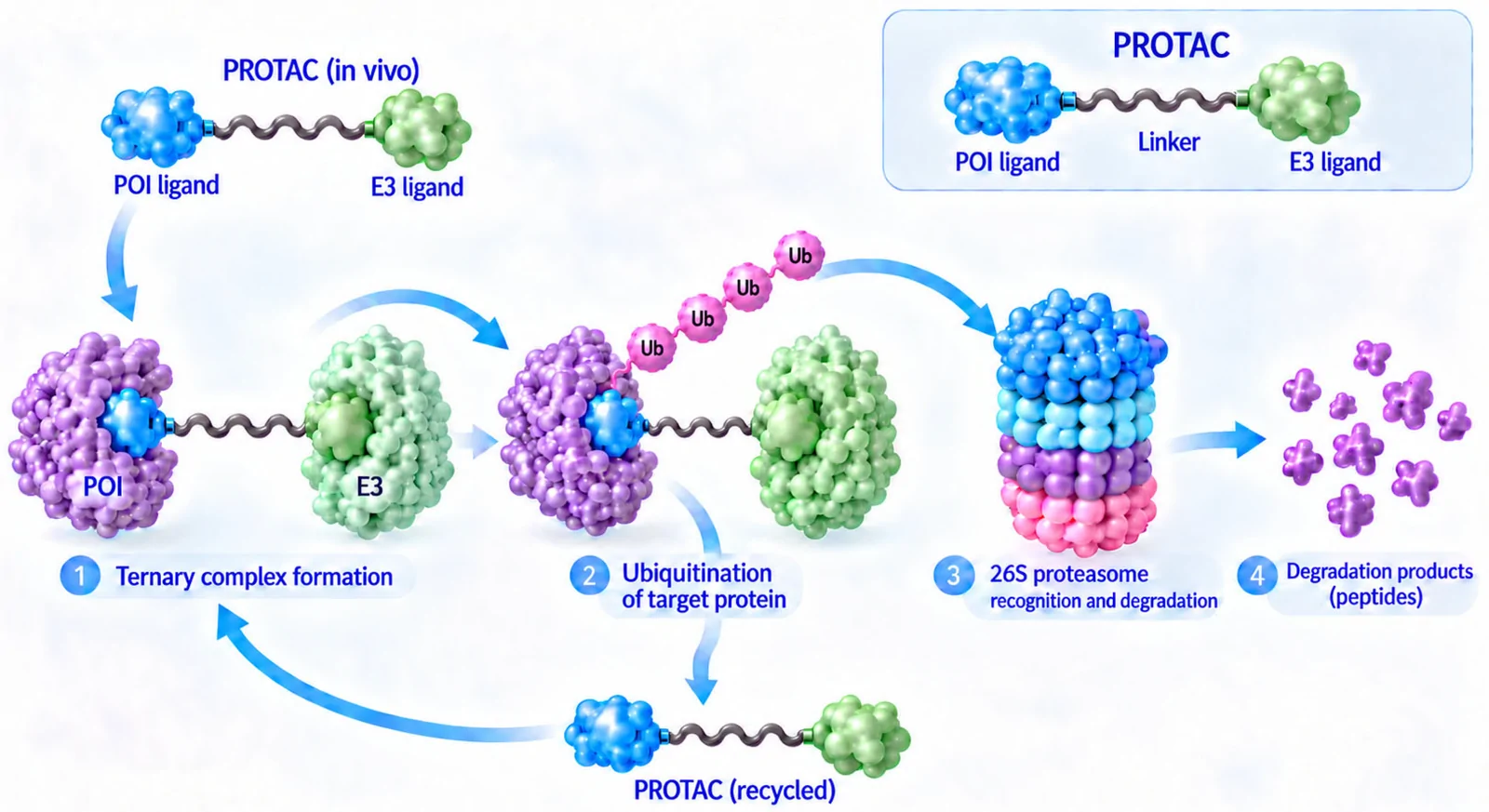
Mechanism of PROTAC-mediated event-driven targeted protein degradation. PROTACs simultaneously bind the target protein and an E3 ligase, forming a ternary complex and inducing polyubiquitination; once the target protein is cleared by the 26S proteasome, the PROTAC can enter the next degradation cycle.

Classical PROTACs consist of three components: a ligand for the protein of interest (POI), a ligand for an E3 ubiquitin ligase, and a linker [9,10]. They induce the formation of a POI–PROTAC–E3 ternary complex, promote POI polyubiquitination and subsequent proteasomal clearance. The degrader can then enter a new degradation cycle [11,12,13,14,15]. The geometry, cooperativity, and residence time of the ternary complex often predict degradation efficiency better than simple binary binding affinity (Figure 1).

### 1.3. Clinical Translation Bottlenecks of Conventional PROTACs

These advantages come with substantial druggability challenges. Most PROTACs occupy chemical space beyond the “rule of five”, with high molecular weight, large polar surface area, and multiple hydrogen-bonding sites. This physicochemical profile restricts membrane permeability and oral absorption [16,17,18,19,20]. Adjusting E3 ligase ligands and linker design can shift pharmacokinetic behavior and tissue selectivity [21,22,23], but systemic administration still confronts multiple barriers. Plasma protein binding, metabolic clearance, non-specific tissue distribution, and off-tumor degradation in healthy tissues all conspire to narrow the therapeutic window [24,25,26,27]. At high concentrations, PROTACs may also be diverted into non-productive binary complexes, producing a bell-shaped concentration–effect relationship known as the hook effect [14,15]. Effective PROTAC optimization, then, depends less on maximizing total tissue exposure and more on ensuring that structurally intact molecules actually reach target cell cytoplasm and persist at concentrations capable of driving productive ternary complex formation.

### 1.4. Definition, Potential Advantages, and Scope of This Review on Self-Assembled Nano-PROTACs

Self-assembled Nano-PROTACs are nanostructures built from degraders themselves, PROTAC prodrugs, amphiphilic conjugates, or complementary functional precursors. These components self-organize through non-covalent interactions or in situ reactions [28,29,30,31,32,33,34]. Driving forces behind this assembly span hydrophobic association, π–π stacking, and hydrogen bonding to electrostatic interactions, host–guest recognition, peptide folding, and bioorthogonal chemistry [35,36,37]. To connect molecular structure with assembly outcomes, researchers now rely on structural prediction, high-throughput screening, and molecular simulations. In contrast to conventional “nanocarrier-encapsulated PROTACs”, the key distinction of self-assembled systems lies in the direct participation of the degrader or its functional modules in structural formation, rather than serving merely as passive cargo. This construction strategy directly couples degradation function with nanostructure formation, enabling carrier-free co-assembly or PROTAC conjugate self-assembly to increase the proportion of functional components and minimize additional variables introduced by large amounts of inert carriers [33,38]. Building on this, the incorporation of endogenous stimuli (such as pH, glutathione [GSH], ROS, enzymes, or hypoxia) or exogenous stimuli (including light, radiation, or ultrasound) enables further regulation of disassembly, bond cleavage, or in situ construction. This allows the release or formation of active degraders within specific tissues, cellular compartments, or stimulus windows, thereby reducing off-target tissue exposure and, in selected systems, mitigating the hook effect [39,40,41,42,43,44,45,46]. In addition, self-assembly enables the integration of protein degradation with complementary mechanisms (such as photodynamic therapy, chemotherapy, radiotherapy, or immunomodulation) within a single particle or local reaction environment. This mitigates the risk of asynchronous in vivo distribution of different components to some extent, and provides a foundation for different therapeutic modules to exert their effects within the same lesion and time window [47,48,49,50,51,52]. These features establish a mechanistic link between self-assembled Nano-PROTACs and the critical needs in tumor therapy: nanoassembly is expected to mitigate the limitations of conventional PROTACs in systemic exposure, tissue distribution, and intracellular delivery, while the abnormal acidic microenvironment, redox state, ROS levels, and specific enzyme expression in tumor tissues can serve as endogenous triggers for the localized release or in situ formation of degraders within the lesion. For targets that are difficult to continuously control with traditional inhibitors—such as drug-resistant associated proteins, aberrant transcription factors, and immunometabolic nodes—self-assembled systems can also enable spatiotemporally confined protein degradation and synergize with chemotherapy, photodynamic therapy, radiotherapy, or immunomodulation, thereby offering new interventional avenues to overcome tumor heterogeneity and therapeutic resistance.

This review adopts the correspondence among “construction strategies, activation features, and levels of evidence” as the primary analytical framework [28,29,30,31,32]. First, the research scope is defined by whether PROTACs directly participate in assembly, and the main construction strategies are categorized based on the building blocks and the modes of Nano-PROTAC formation. Second, different levels of evidence—including tumor enrichment, cytosolic active species, target protein degradation, and antitumor efficacy—are distinguished. Subsequently, the main construction strategies of self-assembled Nano-PROTAC are summarized from the perspective of structural design, and their therapeutic applications are further categorized by disease types—including breast cancer, colorectal cancer, lung cancer, and other cancers. Finally, the clinical translation challenges in structure–assembly–degradation relationships, safety, as well as quality control and scalable manufacturing are discussed.

### 1.5. Literature Search and Scope

This narrative review identified relevant studies published between 2000 and 2026 through PubMed/MEDLINE, using combinations of search terms including “PROTAC”, “self-assembly”, and “nanoassembly”. Primary studies were included when PROTAC-containing building blocks or complementary precursors formed assemblies with evidence of target degradation in cancer-related models. Formulations based solely on physical loading of unmodified PROTACs into separate carriers were excluded. SPNpro and LPN were retained because their PROTAC-containing conjugates constitute assembly building blocks rather than merely encapsulated cargo.

## 2. Biological Barrier Crossing and the “Delivery–Assembly–Degradation–Efficacy” Evidence Chain

**Figure 2:**
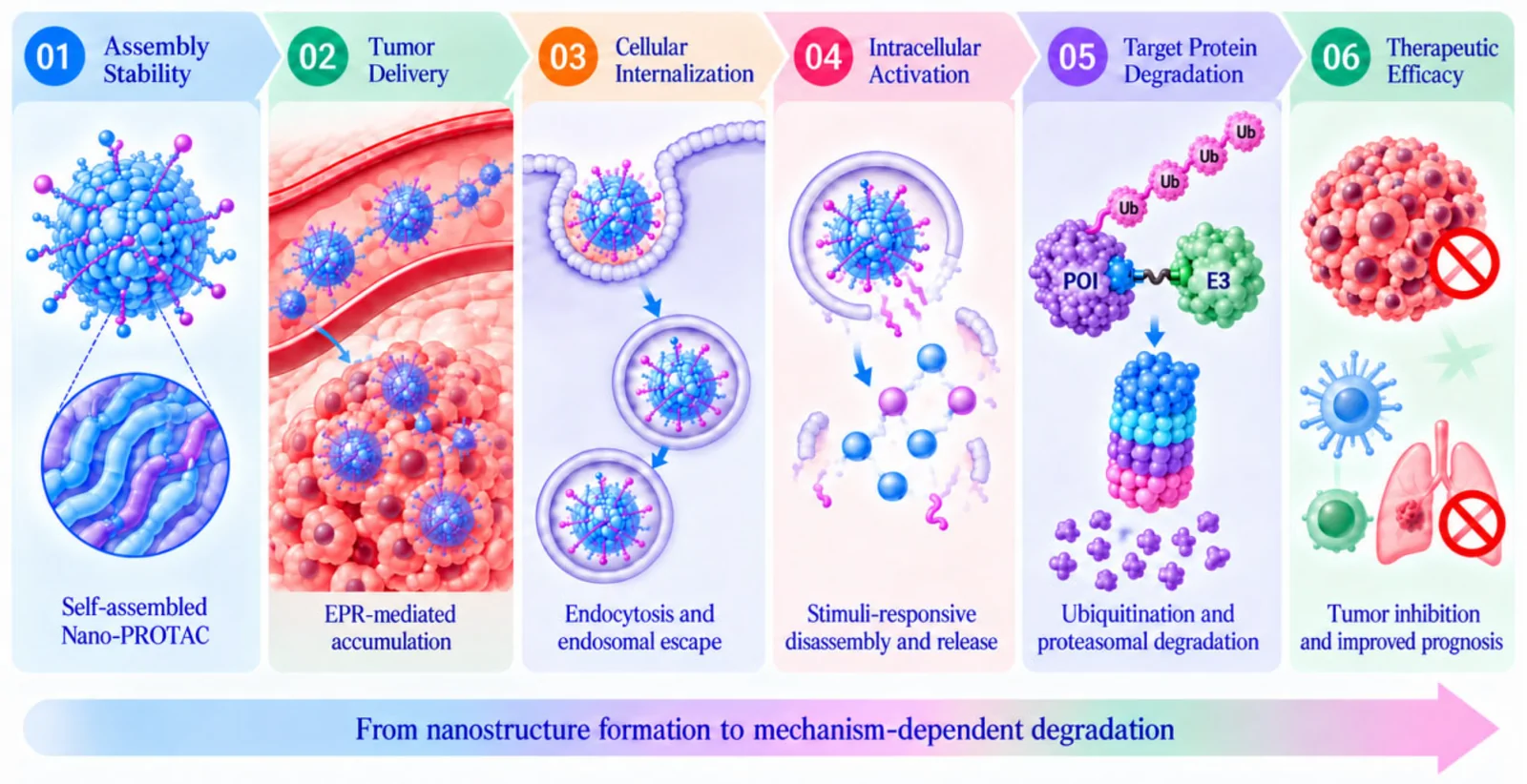
Multistage evidence chain of self-assembled Nano-PROTACs from nanoassembly to mechanism-dependent targeted protein degradation. Self-assembled Nano-PROTACs sequentially undergo assembly stabilization, systemic circulation and tumor delivery, cellular uptake and endosomal escape, release or formation of active degraders in the cytoplasm, POI–PROTAC–E3 ternary complex formation, and in vivo target protein degradation, ultimately exerting antitumor effects. Each stage corresponds to a distinct level of validation. Notably, increased tumor tissue enrichment or cellular uptake can only demonstrate improved delivery, but cannot substitute for mechanistic verification of cytosolic intact active species, E3/proteasome-dependent degradation, and therapeutic efficacy.

The in vivo action of self-assembled Nano-PROTACs is not a single “delivery event”, but rather a tandem process comprising dilution and assembly stability, systemic circulation, tumor exposure, tissue penetration, cellular uptake, endosomal escape, active degrader release/formation, ternary complex formation, and targeted protein degradation. Impairment at any single step can prevent tumor enrichment from being translated into mechanism-dependent protein degradation and therapeutic benefit [53,54,55,56,57]. Figure 2 accordingly deconstructs the in vivo process into independently verifiable evidence nodes. Table 1 summarizes the validation assays, expected readouts, and major confounders for the six stages in Figure 2 (from assembly stability to therapeutic efficacy).

### 2.1. From Systemic Exposure to Cytosolic Delivery

#### 2.1.1. Mechanistic Requirements for Catalytic Degradation

The degradation efficiency of PROTACs is not solely determined by intracellular exposure, but is also influenced by factors such as ternary complex formation, cooperativity, and ubiquitination efficiency [12,13,14,15,58]. Live-cell studies further indicate that the formation kinetics and stability of ternary complexes are closely correlated with degradation potency [15,58]. Therefore, for Nano-PROTACs, enhancing cellular uptake or total intracellular drug amount does not necessarily translate into higher degradation efficiency; the key to delivery optimization lies in improving the effective exposure of degradation-active species in the cytoplasm. For this reason, degradation performance should be judged by more than just cellular uptake or total intracellular drug amount. half-maximal degradation concentration (DC_50_), maximum degradation level (D_max_), degradation kinetics, and post-washout protein recovery offer a more complete picture [15].

The hook effect substantiates this notion. In specific systems, GSH-triggered intracellular fibrillar Nano-PROTACs maintain dose-dependent epidermal growth factor receptor (EGFR) degradation across a broad concentration range through multivalent assembly, providing a paradigm for leveraging self-assembly to modulate the hook effect [41]. However, this phenomenon is structure- and system-dependent, and one should not conclude that self-assembly can universally eliminate the hook effect.

#### 2.1.2. From Tumor Enrichment to Cytosolic Effective Exposure

After intravenous administration, Nano-PROTACs first undergo blood dilution, protein corona formation, complement activation, and phagocytic clearance; subsequently, their tumor delivery is further influenced by factors such as vascular perfusion, endothelial permeability, interstitial pressure, extracellular matrix, and lesion heterogeneity [54,55,56,59,60,61,62]. These processes are highly model-dependent; therefore, tumor enrichment observed in animal studies cannot be simply extrapolated to clinical settings. For instance, rapidly growing mouse xenografts typically exhibit high vascular permeability, and their enhanced permeability and retention (EPR) effect may be stronger than that in human solid tumors; therefore, EPR should not be regarded as a stable and universal tumor-targeting mechanism. More importantly, even if nanoparticles accumulate in tumor tissues, bulk fluorescence or total drug concentrations may still include signals from the intravascular space, extracellular matrix, cell membrane, and endo/lysosomal compartments; therefore, they cannot confirm that intact, degradation-active PROTACs have reached the cytoplasm.

After extravasation, nanoparticles must still undergo deep tissue penetration, cellular uptake, and endosomal escape, followed by the release or formation of degradation-active cytosolic species [63]. Fluorescence tracking can be compromised by factors such as dye detachment, cargo–carrier decoupling, or vesicular entrapment; therefore, relying solely on tissue or cellular fluorescence signals makes it difficult to determine effective cytosolic exposure [64,65]. More rigorous evidence should employ liquid chromatography–tandem mass spectrometry (LC–MS/MS) to distinguish intact PROTACs, conjugates, intermediates, and metabolites, and, where feasible, complement bulk tissue measurements by integrating subcellular fractionation, mass spectrometry imaging, or validated cytosolic reporting systems [27,66,67].

### 2.2. Balancing Assembly Stability and Cytosolic Degradation Activity

Self-assembled Nano-PROTACs need to stay intact long enough to reach their target, but once they get there, they have to let go. Disassembly, bond cleavage, or in situ construction at the right moment is what restores degradation activity. This puts the design up against a tight trade-off: keep the system stable in circulation, but make sure it falls apart on cue once inside a tumor or cell. The sweet spot is a system that holds its cargo in blood, wakes up under physiological stimulus levels, and presents POI- and E3-binding arms in the right spatial arrangement for ternary complex formation.

This balance can be approached through chemically distinct designs. Ma et al. linked an EGFR-targeting PROTAC to FA–PEG through a disulfide bond, coupling micellar delivery with reduction-responsive activation [68]. Zou et al. developed self-assembling polymer–PROTAC prodrugs that release free degraders under reducing conditions [69]. Yang et al. further separated retention from activation: phosphatase-mediated conversion promoted nanofiber formation, whereas subsequent carboxylesterase-mediated hydrolysis liberated ARV-771 [70]. Together, these examples illustrate how cleavable linkages and sequential activation can reconcile assembly mediated delivery with the availability of an active degrader.

Hydrophobic forces, π–π stacking, hydrogen bonding, electrostatics, and solvation collectively dictate how these systems assemble. Work on carrier-free nanodrugs, stimuli-responsive polymers, and non-covalent protein complexes shows that molecular structure, concentration, salts, and serum proteins can push assembly states in markedly different directions [33,34,38,71,72,73]. Linker length, flexibility, and attachment site influence not only molecular packing but also ternary complex geometry [22,23]. Computational tools such as Rosetta, molecular dynamics, deep learning, and high-throughput nanoformulation screening help narrow down the options, but at the end of the day you still need to check assembly morphology, ternary complex formation, and actual degradation readouts experimentally [35,36,74,75,76].

Ligand accessibility constitutes another independent variable. The POI ligand or E3 ligand may be buried within the particle core or sterically hindered by the PEG/hydrophilic corona; biological activity can be restored through complete disassembly, cleavage of labile bonds, or conformational rearrangement. Covalent conjugation systems especially require distinguishing the parent conjugate, intermediates, and released PROTAC, and reporting the degree of substitution or functionalization ratio, as these parameters concurrently influence the hydrophilic–lipophilic balance, ligand density, and batch-to-batch consistency [77,78,79].

Currently, no universally applicable quantitative relationship has been established between particle size, morphology, or surface charge and DC_50_/D_max_. While dimensions and surface chemistry dictate tissue distribution and intracellular trafficking, degradation potency is further determined by cytosolic free concentration, ternary complex cooperativity, ubiquitination efficiency, and protein resynthesis [12,14,15,55,56,57]. Therefore, structure–activity studies should ideally alter a single chemical variable at a time, with concomitant measurements of critical assembly concentration, serum stability, ligand accessibility, cytosolic intact drug, ternary complexes, DC_50_, D_max_, and recovery kinetics.

Based on the above analysis, the following sections will categorize the main construction strategies according to the building blocks and the modes of Nano-PROTAC formation, and further differentiate their activation loci and stimulation modalities, so as to avoid mixing construction modes with stimulus responsiveness within the same classification tier.

## 3. Main Construction Strategies and Activation Features of Self-Assembled Nano-PROTACs

**Figure 3:**
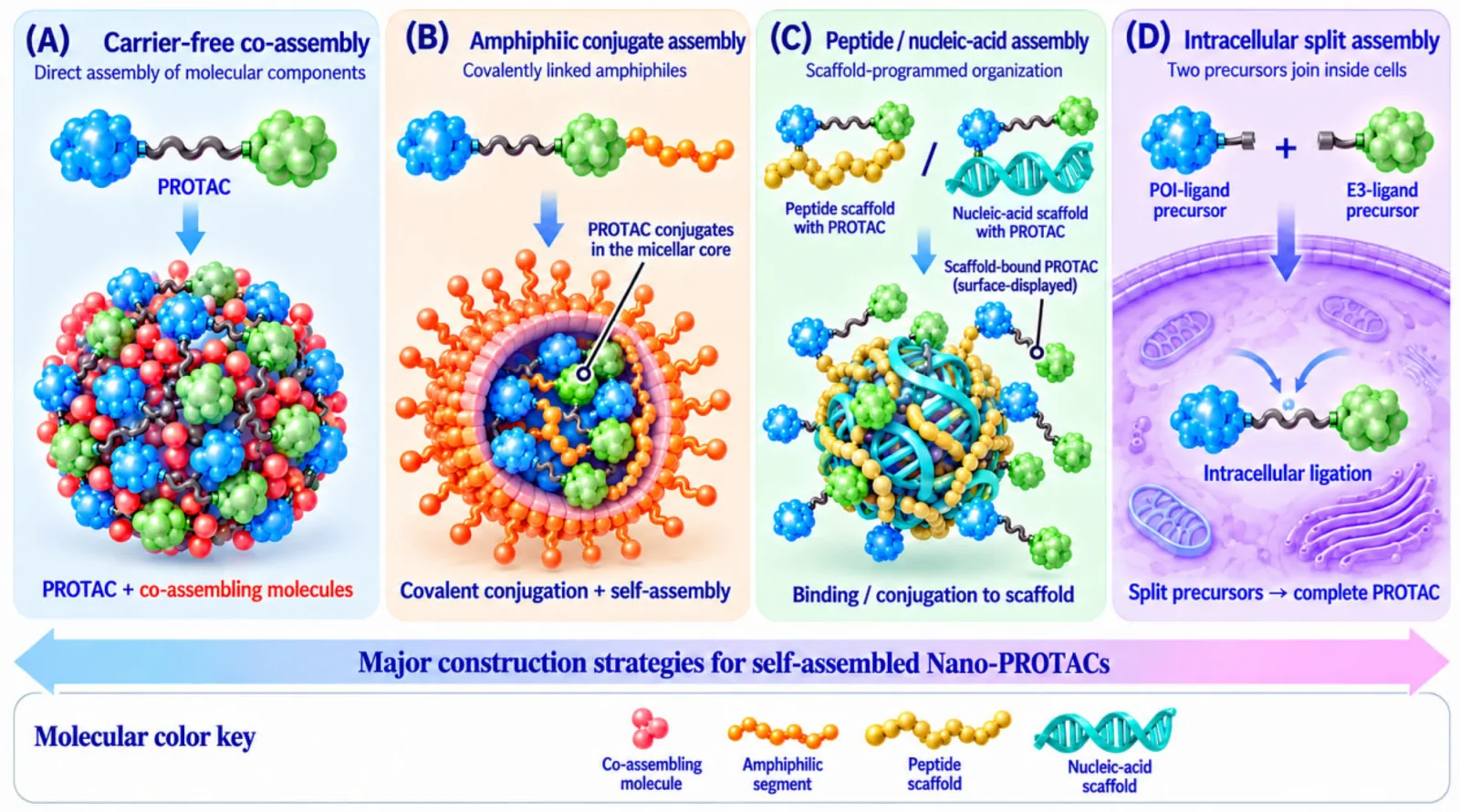
Four main construction strategies of self-assembled Nano-PROTACs. (**A**) Carrier-free co-assembly: PROTACs and therapeutic molecules primarily form nanostructures directly via non-covalent interactions such as hydrophobic interactions, π–π stacking, and hydrogen bonding. (**B**) Amphiphilic conjugate self-assembly: Following covalent linkage of PROTACs to hydrophilic/lipophilic or functional modules, amphiphilic driving forces direct the formation of micelles or nanoparticles. (**C**) Peptide/nucleic acid-programmed assembly: leveraging peptide folding, nucleic acid hybridization, and multivalent molecular recognition to achieve sequence-programmable nano-assembly. (**D**) In situ construction from separate precursors: functional modules are delivered independently and subsequently assembled into active degradation systems at tumor sites or within cells via bioorthogonal reactions or other means. All four strategies must balance circulatory stability, effective cytosolic exposure, and protein degradation activity.

According to the building blocks and the modes of Nano-PROTAC formation, existing self-assembly systems can be classified into four main construction strategies: carrier-free co-assembly of PROTAC building blocks or therapeutic molecules, self-assembly of amphiphilic PROTAC conjugates, peptide/nucleic acid programmed assembly, and intracellular or tumor-localized in situ construction from separate precursors. The four strategies rely on distinct mechanisms:non-covalent intermolecular interactions, amphiphilic driving forces, sequence-encoded molecular recognition and folding, and bioorthogonal reactions, to form active nanosystems. pH, GSH, ROS, enzymes, hypoxia, light, radiation, and ultrasound serve as horizontal activation dimensions that regulate disassembly, release, or in situ construction (Figure 3).

### 3.1. Carrier-Free Co-Assembly

#### 3.1.1. Assembly Modes and Design Principles

Carrier-free co-assembly employs intact PROTACs and a second therapeutic molecule as primary building blocks, directly forming nanostructures via non-covalent interactions (including hydrophobic association, π–π stacking, hydrogen bonding, electrostatic interactions, or host–guest recognition) [33,38,73]. In practice, researchers mix components at set ratios, then shift the solvent environment—through displacement, dilution, or controlled mixing—to trigger nucleation and particle formation. A small amount of phospholipids or PEG lipids can be added if colloidal stability needs a boost. That said, assembly outcomes are exquisitely sensitive to component ratio, concentration, solvent composition, ionic strength, and mixing conditions. Critical assembly concentration (CAC), particle size, PDI, morphology, and serum stability should all be on the checklist when evaluating how a given formulation behaves.

#### 3.1.2. Advantages and Limitations

Carrier-free systems can reduce the use of inert carrier materials and increase the proportion of therapeutic components, but their performance depends on balancing assembly stability with the availability of active species. Park et al. assessed serum colloidal stability, Cat-B-triggered cleavage, and the binding of the released PROTAC to HK2 and VHL [80]. Yue et al. likewise reported plasma stability and pH-responsive release for co-assembled BVT-NPs [81]. These examples illustrate the importance of evaluating formulation stability together with activation or release. We therefore recommend that ligand accessibility, intact drug fraction, serum stability, ternary complex formation, and proteasome/E3 dependency be examined, where applicable, alongside particle size and cellular uptake.

### 3.2. Amphiphilic Conjugate Self-Assembly

#### 3.2.1. Assembly Modes and Design Principles

Amphiphilic PROTAC conjugates are first synthesized by covalently integrating PROTACs with PEG, lipids, polymers, or photosensitizer modules into building blocks with defined hydrophilic–lipophilic partitioning. These building blocks then self-assemble into micelles or nanoparticles in aqueous media driven by hydrophobic effects [22,39,40,82,83]. Assembly can be induced during preparation via solvent displacement, thin-film hydration, or controlled mixing, wherein the hydrophilic–lipophilic balance, linkage site, and functional module ratio collectively determine the final assembly structure. Further incorporation of disulfide bonds, ester bonds, enzyme substrates, or oxidation-sensitive bonds enables the assemblies to remain relatively stable during circulation and undergo cleavage-induced release in pathological or intracellular microenvironments.

#### 3.2.2. Advantages and Limitations

The advantages of covalent amphiphilic conjugates lie in their ability to integrate targeting, stimuli-responsiveness, and combination therapy within a common, nanoplatform, potentially improving the coordination of delivery and activation [68,69,80]. Potential limitations include conjugation heterogeneity in polymeric systems, premature or delayed linker cleavage, and the possible formation of inactive intermediates. Studies should report molecular identity or degree of substitution, as appropriate, together with critical assembly concentration and plasma stability. Beyond that, quantitative methods should track how the conjugate evolves over time—from the parent conjugate through any detectable cleavage intermediates to the released PROTAC.

### 3.3. Peptide and Nucleic Acid-Programmed Assembly

#### 3.3.1. Design Principles

Peptides and nucleic acids do double duty in these systems: they hold the structure together and carry instructions for targeting, stimulus response, and multivalent arrangement. Peptides, for instance, self-assemble into fibrils or nanoparticles driven by β-sheet formation, aromatic amino acid stacking, and enzyme-cleavable sequences. Nucleic acids take a different route—base pairing, aptamer recognition, and DNA nanostructures let researchers program exactly where each functional module sits [84,85,86,87,88,89,90]. For example, DNA tetrahedral PROTACs illustrate the advantage of programmable multivalent organization, although the benefit of a specific architecture cannot be assumed for other valencies or geometries [85]. Aptamer–PROTAC conjugates offer tumor-targeted delivery, but their performance depends on aptamer-mediated uptake and subsequent payload release [86,87]. When the aptamer itself serves as the target-binding module, as in nucleolin degradation, selective recognition must also lead to productive intracellular target–E3 engagement. To construct these systems, sequence–module connections are first defined, followed by assembly through annealing, buffer adjustment, or environmental cues. Overall, rational design requires tuning ligand density, the POI:E3 module ratio, and spatial spacing while ensuring that the resulting architecture supports productive intracellular delivery and degradation.

#### 3.3.2. Advantages and Limitations

Peptide and nucleic acid platforms offer sequence programmability, flexible module swapping, and multivalent design. DNA tetrahedral AS-TD2-PRO improved targeting and degradation through multivalent module presentation, although these benefits remain architecture-dependent [85]. AS1411–PROTAC conjugates improved tumor targeting and tolerability, but their activity also requires effective uptake and linker cleavage [86]. In peptide-based Nano-PROTACs, GSH-triggered intracellular fiber formation supported multivalent degradation networks with an anti-hook effect, highlighting the functional importance of assembly while making stimulus-dependent conversion a prerequisite [41]. These examples show that programmable recognition and organization must translate into productive intracellular delivery and degradation. For nucleic acid-based systems, nuclease degradation, endosomal entrapment, potential immunogenicity, and manufacturing challenges remain important considerations [89]. Evaluation should therefore link sequence integrity, ligand presentation, and serum stability to release or assembly and concentration-dependent activity. Immunotoxicity and repeated-dose clearance require separate assessment.

### 3.4. Separate-Precursor and Intracellular In Situ Construction

#### 3.4.1. Design Principles

Separate-precursor strategies break the POI ligand, E3 ligand, or linker into smaller pieces that are made and delivered on their own. Once inside the cell, tetrazine–trans-cyclooctene (TCO) click chemistry, catalytic deprotection, or other bioorthogonal reactions stitch them back into an intact degrader [91,92,93]. Putting this approach together takes three steps: synthesize the precursors, build a delivery system for them, then trigger the intracellular reaction.

#### 3.4.2. Advantages and Limitations

Separate-precursor systems enable conditional degrader formation or activation, but require both components to meet in a reaction-compatible intracellular environment. Lebraud et al. achieved degradation through sequential precursor treatment, whereas preformed CLIPTACs were inactive, consistent with poor cellular permeability [91]. Chang et al. further demonstrated integrin-dependent activation using an RGD–tetrazine activator [92]. These examples illustrate how reaction location and targeted activation can influence degradation. Hu et al. likewise identified delivery of both components as a limitation and separately characterized ligation products by HPLC and MALDI-TOF-MS [94]. These findings motivate separate assessment of chemical conversion and biological activity: target depletion alone should not be treated as a quantitative measure of intracellular product formation. We therefore recommend that precursor and product measurements complement degradation assays, with compartment-resolved analysis where feasible. Catalyst-only controls are additionally warranted when metal- or photocatalytic activation is used.

### 3.5. Stimuli-Responsiveness and Activation Features Across Construction Strategies

#### 3.5.1. Design Logic

Stimuli-responsiveness does not constitute a fifth category of systems independent of the aforementioned four construction strategies; rather, it represents a horizontal design dimension that regulates the disassembly, release, or in situ construction of Nano-PROTACs at specific spatiotemporal windows. Endogenous triggers include pH, GSH/redox, ROS, cathepsins, MMPs, sulfatases, and hypoxia [95,96,97,98,99,100]; exogenous triggers comprise light, radiation, and ultrasound [39,42,43,44,101,102,103]. Small-molecule endeavors such as photoPROTAC and PHOTAC have laid the chemical foundation for spatiotemporally controlled degradation [104,105,106,107]; design should specify which step (disassembly, bond cleavage, conformational rearrangement, or in situ formation) is regulated by the stimulus, and ensure that the response threshold falls within physiologically or pathologically achievable ranges.

#### 3.5.2. Advantages and Limitations

**Figure 4:**
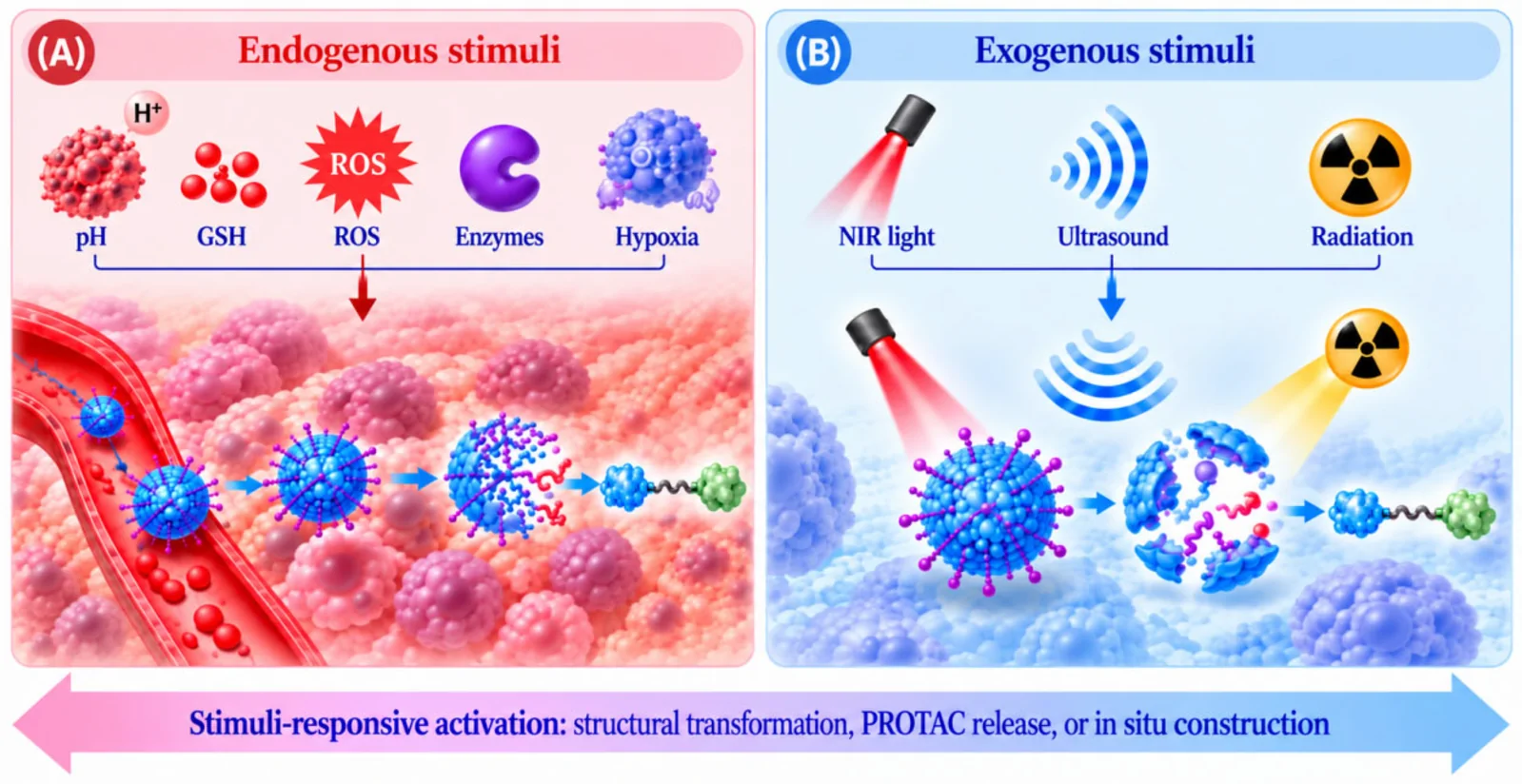
Stimuli-responsiveness as a horizontal activation dimension spanning distinct Nano-PROTAC construction strategies. (**A**) Endogenous stimuli: Exploiting aberrant features of the tumor microenvironment—such as pH, GSH/redox, ROS, enzymes, and hypoxia—to trigger assembly disassembly, linker cleavage, structural rearrangement, or active PROTAC formation. (**B**) Exogenous stimuli: Utilizing external energy sources—such as light, radiation, or ultrasound—to achieve spatiotemporal control over PROTAC release or in situ activation. Stimuli-responsiveness is not an independent construction category, but rather a horizontal regulatory mechanism that endows distinct self-assembling systems with spatiotemporal selectivity.

The primary advantage of stimuli-responsive strategies lies in confining PROTAC activity to specific tissues, cellular compartments, or external stimulation windows, potentially reducing off-target effects while preserving degradation potency and coordinating degradation with other therapeutic modalities. However, clinical translation remains constrained by stimulus heterogeneity, activation thresholds, and, for light-responsive systems, limited tissue penetration [108]. Evaluation should therefore characterize stimulus levels, activation and release kinetics, background activation in normal tissues, and the availability of active intracellular species. Resistance to the hook effect is also system-specific: Zhang et al. reported GSH-triggered Nano-PROTACs with an anti-hook effect, whereas Hu et al. observed a hook effect in their supramolecular degradation platform [41,94]. Claims of hook-effect mitigation should therefore be supported by concentration–degradation curves extending into the high-concentration range, together with non-assembling or assembly disrupted controls where feasible (Figure 4).

## 4. Applications of Self-Assembled Nano-PROTACs in Cancer Therapy

Self-assembled Nano-PROTACs have been investigated in diverse solid-tumor models using strategies such as stimuli-responsive delivery, spatiotemporal activation, and combination therapy. Based on the available literature, this section organizes these applications by tumor type and discusses representative advances in breast, colorectal, and lung cancers, as well as other malignancies.

### 4.1. Self-Assembled Nano-PROTACs for Breast Cancer Therapy

Breast cancer is a complex disease that varies greatly from one case to another and is one of the most common cancers worldwide. TNBC (triple-negative breast cancer), in particular, lacks clearly defined receptor targets and is characterized by drug resistance, high metastatic potential, and a profoundly immunosuppressive tumor microenvironment. These features underscore the urgent clinical need for new therapeutic strategies that precisely target epigenetic regulatory mechanisms and key apoptotic pathways.

Studies of self-assembled Nano-PROTACs in breast cancer have focused primarily on developing stimuli-responsive nanosystems to overcome resistance to apoptosis, regulate epigenetic transcriptional activation, and remodel the immunometabolic tumor microenvironment. To overcome resistance to apoptosis, a sulfatase-responsive Supra-PROTAC assembles into nanofibers within tumor cells and induces Bcl-xL degradation, thereby enhancing apoptosis and chemotherapeutic efficacy [109]. For the epigenetic target BRD4 (bromodomain-containing protein 4), different systems primarily enhance degradation selectivity by controlling where and when the active degrader is generated. Specifically, NAP harnesses singlet oxygen generated by near-infrared irradiation to cleave the linkage and release the active PROTAC, enabling spatiotemporally coordinated protein degradation and photodynamic therapy [49]. A spatially confined nanoplatform sequentially exploits MMP-2-mediated proteolysis, acidic pH, and ROS to regenerate ARV-771; proteasome inhibition experiments further confirmed that the resulting degradation depends on the ubiquitin–proteasome system [110]. A multifunctional POLY-PROTAC further integrates MMP-2-mediated de-shielding, acid-sensitive charge reversal, and reduction triggered by intracellular GSH in sequence. In MDA-MB-231 tumor-bearing mice, rigorous WB, immunohistochemistry (IHC), and carrier-control experiments collectively provided comprehensive evidence of BRD4 degradation [111]. Moreover, the target spectrum has expanded to include immunometabolic and transcriptional regulation. SPNpro degrades IDO to modulate tryptophan–kynurenine metabolism and enhance photodynamic immunotherapy [112]. The peptide prodrug NFTP harnesses active targeting through α6 integrin and in situ assembly to degrade the otherwise undruggable transcription factor FOXM1 and suppress cancer stemness in TNBC [113].

### 4.2. Self-Assembled Nano-PROTACs for Colorectal Cancer Therapy

CRC (colorectal cancer) is a common malignancy of the gastrointestinal tract. Its development and progression are often accompanied by enhanced aerobic glycolysis, formation of a lactate-rich immunosuppressive microenvironment, cell-cycle dysregulation, and reciprocal compensation among multiple oncogenic signaling pathways. Conventional immune checkpoint blockade elicits very low response rates in “cold” tumors such as MSS (microsatellite-stable) CRC.

Given the complex pathology of CRC, research on self-assembled Nano-PROTACs has increasingly focused on the coordinated targeting of metabolic reprogramming, cell-cycle dysregulation, and immunosuppressive networks. For coordinated metabolic–immune regulation, carrier-free DdLD NPs are formed by the coassembly of DOX (doxorubicin) and a BRD4 degrader. Chemotherapy-induced immunogenic cell death and inhibition of the BRD4/c-Myc axis jointly modulate tumor metabolism and the immune microenvironment [50]. For direct targeting of immune molecules, self-assembling peptide-based PT-NPs comprise a recognition sequence for PD-L1 (programmed death-ligand 1), a self-assembling FF dipeptide motif, and an E3 ligase-recruiting peptide. Following receptor-mediated endocytosis, they accumulate in tumor cells and promote the degradation of cell-surface and intracellular PD-L1 via lysosomes and the cytosolic UPS (ubiquitin–proteasome system). The resulting loss of PD-L1 enhances antitumor T-cell responses [114]. To address the interconnected resistance networks in colorectal cancer, NP-PROTACs employ a dual-targeting design. Peptide PROTACs targeting β-catenin and STAT3, respectively, are conjugated to phospholipids through disulfide bonds and self-assemble into reduction-responsive micelles. In HCT116 and CT26 models, dual degradation of β-catenin and STAT3 further suppressed compensatory survival pathways. This degradation was accompanied by increased infiltration of CD103^+^ dendritic cells and activation of T cells with specific cytotoxic activity [115]. IDO degradation further couples immunometabolic modulation with local PDT (photodynamic therapy). In the CT26 model, cathepsin B cleaves the peptide linker of the carrier-free prodrug assembly LPN, releasing an active PROTAC. This promotes IDO degradation and inhibits kynurenine metabolism. Together with the in situ vaccine effect induced by local PDT, these combined effects suppressed primary tumor growth, distant metastasis, and postoperative recurrence in this model [116]. Taken together, these CRC studies do not focus on a single oncogenic protein. Instead, these strategies reshape the tumor microenvironment by degrading regulators of metabolism, immune responses, and compensatory signaling. The resulting changes in local metabolic and immune conditions also restrict alternative pathways that could blunt their effects.

### 4.3. Self-Assembled Nano-PROTACs for Lung Cancer Therapy

Non-small cell lung cancer (NSCLC) has a high mortality rate, and its treatment relies heavily on inhibitors targeting driver kinases such as EGFR and anaplastic lymphoma kinase (ALK). Secondary mutations, including EGFR T790M and C797S, frequently cause acquired resistance to targeted therapy. In addition to the hook effect, conventional small-molecule PROTACs face further in vivo limitations, particularly poor drug-like properties.

Studies of self-assembled Nano-PROTACs for lung cancer have focused on driver mutation-associated resistance, the hook effect in certain systems, and DNA damage repair in tumor cells. The GSH-responsive Anti-hook Nano-PROTAC assembles into multivalent nanofibers in the cytoplasm. These nanofibers sustain EGFR degradation over a broad concentration range and reduce the hook effect at high concentrations [41]. The self-assembling peptide EGFR NanoTAC targets wild-type EGFR and drug-resistant EGFR mutants [117]. The carrier-free construct SDNpro uses a different approach. It combines BRD4 degradation with DNA damage induced by photodynamic therapy and increases antitumor efficacy [47]. These studies test protein degradation against concentration-dependent loss of activity and resistance mutations. They also examine sensitization through combination therapy.

### 4.4. Self-Assembled Nano-PROTACs for the Treatment of Other Cancers

Self-assembled Nano-PROTACs have also been tested in models of bladder cancer and castration-resistant prostate cancer (CRPC). These studies do not limit degradation to canonical oncoproteins. They also target molecules involved in ferroptosis defense and resistance associated with hormone-receptor signaling.

In human bladder cancer research, Zhang and colleagues designed a self-assembling Nano-PROTAC from scratch, incorporating a hexameric linker and targeting GPX4 (glutathione peroxidase 4), a difficult-to-drug protein central to ferroptosis defense. The platform combines cell-surface EGFR recognition with cleavage triggered by elevated intracellular ROS in a dual-gating mechanism, and its self-assembled hexameric structure increased plasma stability by up to 48-fold. In the EGFR-high EJ-1 model of human bladder transitional cell carcinoma, it induced tumor-specific GPX4 degradation and lethal lipid peroxide accumulation while reducing systemic toxicity in nontarget tissues [118]. In advanced prostate cancer, castration resistance often stems from AR-V7, a splice variant of AR (androgen receptor), and compensatory expression of molecular chaperones. To address this, Yang and colleagues designed Psa-AR, a nanopeptide targeting PSMA (prostate-specific membrane antigen) that self-assembles in vivo; after entering drug-resistant 22Rv1 prostate cancer cells, it forms β-sheet nanofibers and simultaneously induces degradation of AR (80%), AR-V7 (74%), and HSP90 (65%), thereby significantly prolonging median survival by 15 days in a resistant xenograft model compared with that achieved using a combination of conventional inhibitors [119].

### 4.5. Shared Features and Modes of Action Across Tumor Types

**Figure 5:**
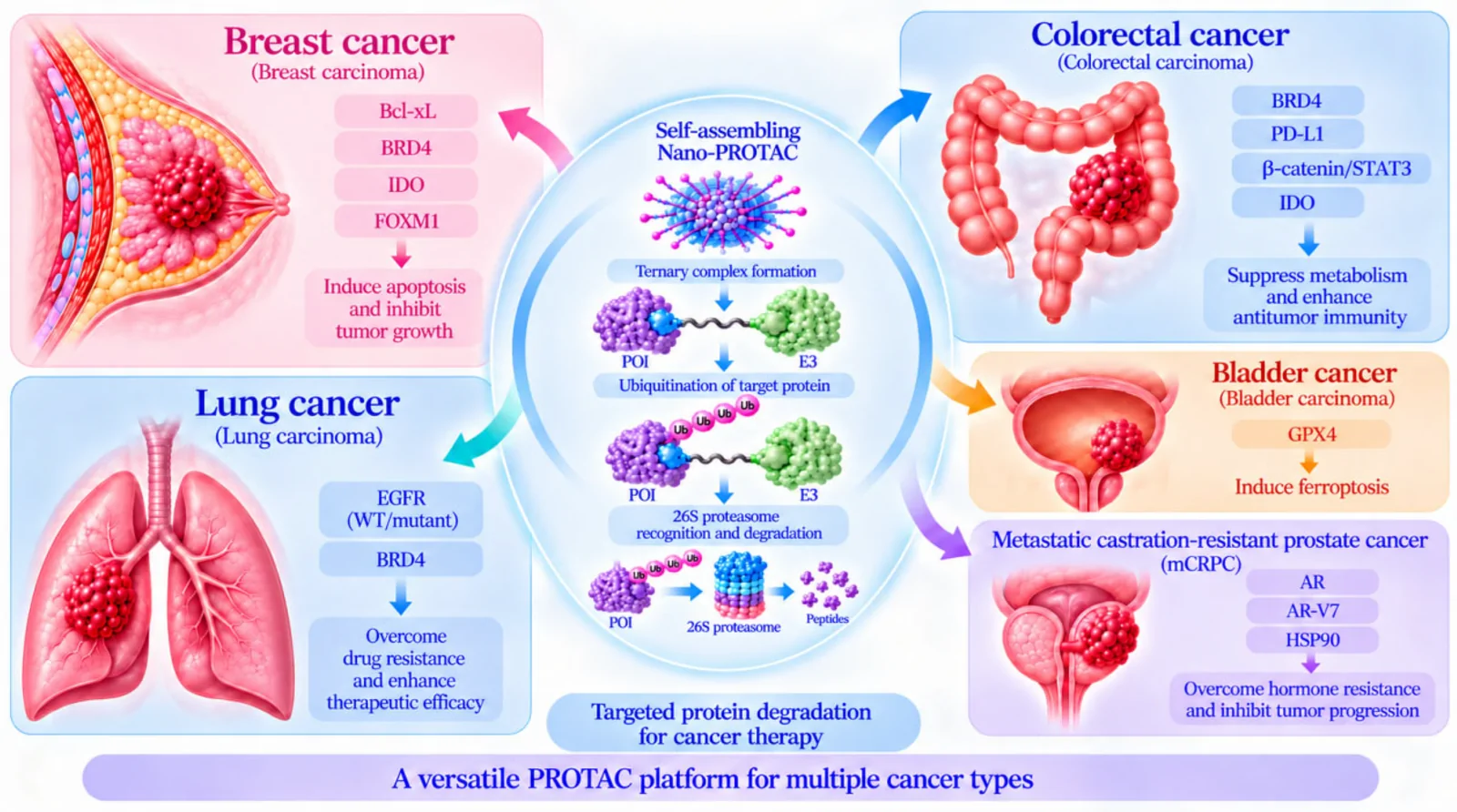
Modes of action and mechanisms of multimodal synergy of self-assembled Nano-PROTACs in cancer therapy. The figure summarizes tumor delivery, stimulus-responsive activation, E3-dependent target-protein degradation, and multimodal therapeutic synergy involving self-assembled Nano-PROTACs. Representative targets shown include BRD4, EGFR, and AR/AR-V7.

Overall, the applications of self-assembled Nano-PROTACs across tumor types appear to be shaped, at least in part, by disease-specific therapeutic needs. Breast cancer studies have mainly focused on the regulation of apoptosis, epigenetic targets, photodynamic therapy, and microenvironment-responsive strategies; colorectal cancer studies have more often addressed metabolic reprogramming and regulation of the immune microenvironment; lung cancer studies have emphasized EGFR resistance mutations, the hook effect, and DNA damage repair; and studies in other tumor models have extended the target spectrum to molecules involved in ferroptosis defense and hormone receptor-associated resistance. Although these approaches differ across tumor types in target selection, construct design, and activation conditions, they share a therapeutic sequence: nanoassembly mediated delivery to lesions, stimulus-responsive disassembly or in situ reconfiguration to generate active cytosolic species, and subsequent mechanism-dependent degradation of target proteins that produces therapeutic effects (Figure 5). Notably, validation of the links in this evidence chain remains uneven: reduced target-protein levels in vivo and antitumor effects have been reported for most systems, whereas direct evidence for intact active species in the cytosol, in vivo E3 dependence, and PK–PD (pharmacokinetics–pharmacodynamics) relationships for degradation remains relatively limited. Accordingly, evaluation of self-assembled Nano-PROTACs should not rely solely on tumor accumulation or end-point tumor growth inhibition, but should establish a continuous evidence chain from delivery to lesions and effective cytosolic exposure to target-protein degradation and mechanistic validation. Table 2 summarizes the design strategies, activation features, in vivo protein-level readouts, mechanistic controls, and safety evidence for representative systems across tumor types.

## 5. Challenges and Clinical Translation

Self-assembled Nano-PROTACs remain at the preclinical proof-of-concept stage, with evidence derived mainly from cell-based studies and mouse tumor models. Beyond antitumor activity, clinical translation will require defining structure–assembly–degradation relationships, linking effective cytosolic exposure to PK–PD, and addressing repeat-dose safety, quality control, and scalable manufacturing.

### 5.1. Structure–Activity Relationships Between Assembly Architecture and Degradation Activity

Once in circulation, nanoparticles encounter dilution, changes in ionic composition, and protein corona remodeling. Consequently, a single-time-point DLS (dynamic light scattering) measurement in pure water cannot capture their state under biologically relevant conditions [59,60,61,62]. At a minimum, studies should report molecular identity and purity, drug content, component ratios, critical assembly concentration, hydrodynamic diameter, PDI, zeta potential, and particle morphology determined by electron microscopy. Time-resolved stability should also be assessed in serum-containing media [77,78]. A self-assembled structure may become kinetically trapped, so evidence is required before it is treated as a system at thermodynamic equilibrium. Microfluidic screening supports formulation exploration [120], while high-throughput screening and machine learning can reveal nanomedicine structure–activity relationships [36]. Molecular dynamics can elucidate assembly behavior [35], and structural descriptors can predict PROTAC nanoparticle formation [37]. MELD-accelerated molecular dynamics additionally supports ligand–protein binding-pose selection [121]. These approaches alone do not establish Nano-PROTAC degradation activity. Following cellular uptake, the assembly must release a cytosol-accessible degrader capable of binding the target and recruiting an E3 ligase. Degradation should be verified through concentration-response and time-course experiments, with controls distinguishing degrader-induced protein loss from nonspecific cytotoxicity or altered protein expression.

Evidence for the proposed mechanism should track the degrader from particle uptake through to target degradation. Uptake assays must show that particles enter cells rather than simply adsorb to the cell surface. The next step is to establish endosomal escape and detect a chemically intact, active degrader in the cytosol, whether it is released from the particle or formed intracellularly. Combining imaging with subcellular fractionation and chemical analysis can reveal the timing and dose dependence of these steps. Appropriate controls are also needed to rule out extracellular release, endosomal trapping, or effects caused by degradation products. It should also include reduced POI levels, rescue by proteasome or NEDD8-pathway inhibition, dependence on the recruited E3, non-binding and inactive controls, target-protein ubiquitination, and recovery kinetics after washout [12,14,15,58]. For multivalent or dual-target assemblies, proteome-wide mass spectrometry should be prioritized to assess selectivity.

### 5.2. Safety and Mechanistic Risks

Safety risks stem from both the material itself and the way it induces degradation. The material may activate complement, trigger infusion reactions, accumulate in phagocytes, damage the liver or spleen, elicit immune responses, or remain in the body over the long term. The degradation mechanism may also affect target proteins in normal tissues, leading to on-target/off-tumor toxicity. Other concerns include E3 ligand-associated neosubstrate effects and disruption of proteostasis [24,25,122]. Assessing body weight and routine serum biochemistry alone is not enough to establish safety. Repeat-dose studies should also assess hematology, complement and cytokine responses, histopathology, POI degradation in major organs, immune phenotypes, and recovery after treatment cessation. For split-component systems, the precursors, catalyst, in situ product, and by-products must each be assessed separately. Resistance and E3 pathway remodeling may also alter long-term toxicity and efficacy [123,124,125].

### 5.3. Quality Control and Scalable Manufacturing

Clinical translation will require establishing a QTPP (quality target product profile) and linking critical material attributes to degradation performance. Candidate critical quality attributes include molecular identity, degree of substitution and component ratios, the fraction of active drug, particle size and morphology, PDI, surface composition, and critical assembly concentration. They also include serum stability, response threshold, release kinetics, sterility, endotoxin levels, residual solvents, and batch-to-batch reproducibility [77,78,79,126,127]. Microfluidic or controlled mixing may be better suited to scale-up than manual solvent displacement, but scale-up changes mixing time, supersaturation, and nucleation. Recovery of particle morphology and degradation activity after lyophilization and reconstitution should also be verified. Experience with liposomal PK and nanomedicine scale-up in carrier-based proximity platforms may guide process development. However, it cannot replace determination of the critical assembly threshold or quality control of degradation activity for self-assembled PROTACs [4,128].

## 6. Outlook

Self-assembled Nano-PROTACs offer a new avenue for addressing the developability bottlenecks and clinical translation barriers of targeted protein degradation. However, advancing from laboratory proof of concept to clinical application will require further work, particularly in three areas:

### 6.1. Establishing Predictive Structure–Activity Relationships Linking Molecular Structure and Self-Assembly Behavior to Cytosolic Degradation Activity

Future research should use high-throughput screening, all-atom MD (molecular dynamics) simulations and machine-learning algorithms to develop models that predict how chemical topology influences particle formation during self-assembly. Models could incorporate PROTAC hydrophobic surface area, aromatic stacking density, conformational flexibility, linker length, and solvation free energy. Relevant training labels include CAC, serum stability under physiological conditions, and intracellular disassembly and release kinetics [35,36,74,75,76]. Negative cases in which stable particles form without intracellular release must be represented in the database. Performance evaluation needs to measure effective cytosolic exposure and catalytic degradation activity, rather than particle formation alone.

### 6.2. Expanding the E3 Ligase Repertoire and Refining Patient Stratification Through Molecular Pathology

The prevailing reliance on CRBN and VHL may restrict tissue selectivity and increase susceptibility to resistance involving the recruited ubiquitination machinery [123,124,125]. For self-assembled Nano-PROTACs, changing the E3 recruiter or linker also changes the molecular building block and may alter hydrophobicity, intermolecular interactions, assembly stability, and intracellular availability. Structural-descriptor-based prediction of PROTAC nanoparticle formation highlights the connection between molecular properties and assembly [37]. Zou et al. further showed that modifying the VHL-binding hydroxyl group with a reduction-sensitive spacer allowed recovery of degradation activity, whereas a noncleavable analogue showed little activity [69]. Thus, expanding the E3 repertoire requires simultaneous evaluation of assembly, chemical activation, and retained target–E3 engagement: improved particle formation is insufficient if the assembled or released species cannot support degradation. Single-cell RNA sequencing and spatial proteomics could guide POI–E3 expression mapping, but productive engagement requires verification after delivery. Responsive designs should likewise match activation thresholds and release kinetics to tissue-relevant GSH, ROS, protease, and oxygen conditions [39,45,46,99].

### 6.3. Rationalizing Combination Therapies: Linking Co-Assembly and Release Kinetics to Synergy and CMC Control

Combining protein degradation with other treatments requires control over co-assembly composition, nanoparticle stability, and activation timing. Zou et al. incorporated doxorubicin into polymer–PROTAC nanoparticles [69], whereas Park et al. linked an HK2-targeting PROTAC and a photosensitizer through a cathepsin-B-cleavable peptide, enabling enzymatic activation before photoirradiation [80]. These designs illustrate why incorporated component ratios and activation kinetics must be related to treatment scheduling. Premature leakage or differential release may separate the agents spatially or temporally, whereas overly stable assemblies may limit active-degrader availability and prevent target depletion before the partner treatment. Studies should therefore distinguish feed from incorporated ratios and connect serum stability and component-resolved release with intracellular exposure, degradation kinetics, and therapeutic response. Improved efficacy alone does not establish synergy; dose-matrix experiments using appropriate Bliss, ZIP, or HSA models should support such claims [129]. Proteasome-inhibition rescue and immune-cell depletion should separately assess mechanistic contributions. Component ratios, conjugation identity, particle-size distribution, stability, and release profiles should also inform CMC specifications, with unnecessary modules removed to improve batch reproducibility and translational feasibility [4,126,127,128].

## 7. Conclusions

Self-assembled Nano-PROTACs now encompass four main design strategies: carrier-free co-assembly, amphiphilic-conjugate self-assembly, programmed peptide/nucleic acid assembly and split-component assembly within cells. Their applications have expanded to models of breast, colorectal, lung, bladder and prostate cancer. Existing studies show that these systems differ substantially in their building blocks, assembly driving forces, activation conditions and therapeutic targets. Yet their efficacy depends on a continuous sequence linking assembly stability and delivery to lesions with active-species formation in the cytosol and target-protein degradation through the intended mechanism. Further development of self-assembled Nano-PROTACs should not focus solely on structural complexity or tumor accumulation. Greater emphasis on predictable structure–assembly–degradation relationships, robust in vivo mechanistic evidence and scalable quality-control systems could support their progression from proof of concept towards clinical translation.

## Figures and Tables

**Table 1:** Validation assays, expected readouts, and major confounders for the six stages in Figure 2.

Stage	Representative Assays	Expected Readouts	Major Confounders
01 Assembly Stability	DLS/TEM; serum/dilution challenge	Retained size/PDI and morphology	Protein corona; aggregation
02 Tumor Delivery	Tissue drug quantification; vessel-marked imaging	Tumor exposure; extravascular distribution	Vascular heterogeneity; label leakage
03 Cellular Internalization	Flow cytometry; endosomal/cytosolic localization	Internalized amount; extravesicular distribution	Surface signal; endosomal trapping
04 Intracellular Activation	Trigger controls; structural/integrity analysis; HPLC/LC–MS	Disassembly; active-drug release	Premature release; probe artifacts
05 Target Protein Degradation	POI ubiquitination; quantitative WB; pathway blockade	Ubiquitination ↑; pathway-dependent protein ↓	Transcriptional suppression; nonspecific protein loss
06 Therapeutic Efficacy	Tumor/metastasis assays; inactive-degrader and component controls	Tumor/metastasis inhibition; degradation contribution	Ligand inhibition; combination effects

Notes: These are representative validation endpoints, not findings established for every formulation. Abbreviations: DLS, dynamic light scattering; TEM, transmission electron microscopy; PDI, polydispersity index; POI, protein of interest; WB, Western blot; HPLC, high-performance liquid chromatography; LC–MS, liquid chromatography–mass spectrometry.

**Table 2:** Design strategies and preclinical evidence for representative self-assembled Nano-PROTACs across tumor types.

Tumor	System/Target	Strategy	Activation/Targeting	Model	In Vivo Readout	Mechanistic Evidence	Safety	Reference
Breast cancer	Supra-PROTAC/Bcl-xL	Programmed peptide/nucleic-acid assembly	Sulfatase; fibers	Breast tumor	Bcl-xL ↓	Enzyme; apoptosis	Tolerated with chemotherapy	[109]
	NAP/BRD4	Amphiphilic-conjugate self-assembly	NIR/^1^O_2_ release	MCF-7	BRD4 ↓	Light/dark controls	Preliminary tolerability	[49]
	PGDAT@N/BRD4	Amphiphilic-conjugate self-assembly	MMP-2/pH/ROS/hypoxia	MDA-MB-231	BRD4 ↓	MG132; WB/IHC	No overt organ injury	[110]
	SPNpro/IDO	Amphiphilic-conjugate self-assembly	CatB release; NIR-PDT	4T1	IDO ↓; Trp–Kyn	CatB; light controls	Preliminary tolerability	[112]
	POLY-PROTAC/BRD4	Amphiphilic-conjugate self-assembly	MMP-2/pH/GSH	MDA-MB-231	BRD4 ↓	WB/IHC; drug/carrier controls	No overt organ injury	[111]
	NFTP/FOXM1	Programmed peptide/nucleic-acid assembly	Integrin α6; activation	4T1	FOXM1 ↓	Protein; negative controls	Low systemic toxicity	[113]
	RPG7/BRD4; RPGD43/CDK9	Amphiphilic-conjugate self-assembly	NRP-1; reduction	MDA-MB-231	BRD4 ↓/CDK9 ↓	Cleavage; DOX combination	No overt toxicity	[69]
	HK2 NanoTAC/HK2	Amphiphilic-conjugate self-assembly	CatB; PDT	Orthotopic 4T1	HK2 ↓	Metabolism; pyroptosis	No overt toxicity	[80]
Colorectal cancer	DdLD NPs/BRD4	Carrier-free co-assembly	Co-delivery	CT26	BRD4 ↓; c-Myc/PD-L1 ↓	Glycolysis; ICD	No overt systemic effects	[50]
	PT-NPs/PD-L1	Programmed peptide/nucleic-acid assembly	PD-L1 uptake	Colon tumor	PD-L1 ↓	Lysosome; UPS	Preliminary biocompatibility	[114]
	NP-PROTACs/β-catenin, STAT3	Amphiphilic-conjugate self-assembly	Disulfide reduction	HCT116/CT26 †	β-catenin ↓; STAT3 ↓	WB/IHC; single-target controls	Limited normal-cell effects; H&E	[115]
	LPN/IDO	Amphiphilic + carrier-free	CatB release; PDT	CT26	IDO ↓; Kyn/Trp ↓	UPS inhibition; light controls	Tolerability: blood/H&E	[116]
Lung/prostate cancer	Anti-hook Nano-PROTAC/EGFR, AR	Programmed peptide/nucleic-acid assembly	GSH; fibers	A549/LNCaP	≤79% ‡	Dose response; assembly controls	Routine assessment	[41]
Lung/colon cancer	EGFR NanoTAC/EGFR	Programmed peptide/nucleic-acid assembly	EGFR uptake	Lung/colon tumors	WT ~95%; mutant ~80% ‡	Lysosome; proteasome	No overt systemic toxicity	[117]
Lung cancer	SDNpro/BRD4	Carrier-free co-assembly	Ce6/dBET57; PDT	H1299	BRD4 ↓	PDT/degradation controls	Weight; histology	[47]
	BVT-NPs/ALK	Carrier-free co-assembly	27B/VAP/TPGS; pH	H3122; mice †	—	ALK WB; release	—	[81]
Bladder cancer	Hexamer-linker Nano-PROTAC/GPX4	Carrier-free/assembling linker	EGFR; ROS	EJ-1; mice	GPX4 ↓	MG132/NAC; stability ×48	Lower off-target toxicity	[118]
Prostate cancer	Psa-AR/AR, AR-V7, HSP90	Programmed peptide/nucleic-acid assembly	PSMA; assembly	22Rv1	AR/HSP90 ↓	Tumor IF; inhibitors	Well tolerated	[119]
Other solid tumors §	FA–PEG–PROTAC/EGFR	Amphiphilic-conjugate self-assembly	Folate; disulfide	Xenograft	EGFR ↓	Targeting; reduction	—	[68]
Other solid tumors §	NapYp-ARV/BRD4	Amphiphilic; peptide assembly	Phosphatase → esterase	Tumor-bearing mice	BRD4 ↓	Assembly → release	Blood/tissue compatibility	[70]

Notes: ↓, reduced protein level; —, not listed here (not proof of absence). Safety is limited to reported experimental conditions. † Cellular and animal models are distinct. ‡ Values are condition-specific; EGFR mutant: L858R/T790M. § Tumor lineage is not specified here. Blue: carrier-free; green: amphiphilic conjugates; orange: programmed peptide/nucleic-acid assembly. Abbreviations: CatB, cathepsin B; GSH, glutathione; ICD, immunogenic cell death; IF, immunofluorescence; NIR, near-infrared; PDT, photodynamic therapy; ROS, reactive oxygen species; UPS, ubiquitin–proteasome system; WB/IHC, Western blot/immunohistochemistry; H&E, hematoxylin and eosin.

## Data Availability

No new data were created or analyzed in this study. Data sharing is not applicable to this article.
